# Metabolomics and Proteomics of *Brassica napus* Guard Cells in Response to Low CO_2_

**DOI:** 10.3389/fmolb.2017.00051

**Published:** 2017-07-25

**Authors:** Sisi Geng, Bing Yu, Ning Zhu, Craig Dufresne, Sixue Chen

**Affiliations:** ^1^Plant Molecular and Cellular Biology Program, University of Florida Gainesville, FL, United States; ^2^Department of Biology, Genetics Institute, University of Florida Gainesville, FL, United States; ^3^Key Laboratory of Molecular Biology of Heilongjiang Province, College of Life Sciences, Heilongjiang University Harbin, China; ^4^Engineering Research Center of Agricultural Microbiology Technology, Ministry of Education, Heilongjiang University Harbin, China; ^5^Proteomics and Mass Spectrometry, Interdisciplinary Center for Biotechnology Research, University of Florida Gainesville, FL, United States; ^6^Thermo Fisher Scientific West Palm Beach, FL, United States

**Keywords:** metabolomics, proteomics, *Brassica napus*, guard cells, low CO_2_, phytohormone

## Abstract

Stomatal guard cell response to various stimuli is an important process that balances plant carbon dioxide (CO_2_) uptake and water transpiration. Elevated CO_2_ induces stomatal closure, while low CO_2_ promotes stomatal opening. The signaling process of elevated CO_2_ induced stomatal closure has been extensively studied in recent years. However, the mechanism of low CO_2_ induced stomatal opening is not fully understood. Here we report metabolomic and proteomic responses of *Brassica napus* guard cells to low CO_2_ using hyphenated mass spectrometry technologies. A total of 411 metabolites and 1397 proteins were quantified in a time-course study of low CO_2_ effects. Metabolites and proteins that exhibited significant changes are overrepresented in fatty acid metabolism, starch and sucrose metabolism, glycolysis and redox regulation. Concomitantly, multiple hormones that promote stomatal opening increased in response to low CO_2_. Interestingly, jasmonic acid precursors were diverted to a branch pathway of traumatic acid biosynthesis. These results indicate that the low CO_2_ response is mediated by a complex crosstalk between different phytohormones.

## Introduction

Guard cells are specialized pairs of cells forming stomata, the tiny pores on leaf surfaces. Stomatal movement in response to various environmental signals as well as endogenous phytohormones such as abscisic acid (ABA) controls the balance of water transpiration and CO_2_ intake for photosynthesis. It plays an essential role for plants to respond and adapt to their changing environmental conditions. An elevation of either intercellular CO_2_ concentration (C_i_) or atmospheric CO_2_ concentration (C_a_) can induce the closure of stomata (Engineer et al., 2015). In contrast, a decrease of CO_2_ concentration will induce the opening of stomata (Negi et al., 2014). Atmospheric CO_2_ has been increasing (at an average rate of about 2.1 ppm per year in the past 10 years (https://www.esrl.noaa.gov/gmd/ccgg/trends/gr.html). In spite of the overall increasing trend, diurnal and periodic decreases in CO_2_ concentration have been observed (Zhu and Yoshikawa-Inoue, 2015). A better understanding of how plants respond to CO_2_ changes is important for us to enhance crop production under the changing environment. The signaling pathway for elevated CO_2_ induced stomatal closure shares many elements with the well-studied ABA signaling pathway in guard cells, including the activation of OPEN STOMATA1 (OST1) and its target SLOW-TYPE ANION CHANNEL 1 (SLAC1) (Xue et al., 2011), reactive oxygen species (ROS) and nitric oxide (NO) production (Shi et al., 2015), and increase of cytosolic Ca^2+^ concentration (Schwartz et al., 1988; Webb et al., 1996). However, the high CO_2_ signaling pathway has its unique components and does not completely overlap with the ABA signaling pathway. For example, several Arabidopsis mutants that are specifically insensitive to elevated CO_2_ have been identified, including carbonic anhydrase double mutant *ca1ca4* (Hu et al., 2010), *rhc1* (*resistant to high CO*_2_*1*) (Tian et al., 2015) and *ht1* (*high temperature 1*) (Tian et al., 2015). A comprehensive review on elevated CO_2_ signaling in guard cells has been published recently (Engineer et al., 2015). Briefly, the signaling pathway starts with the conversion of CO_2_ to bicarbonate by the activity of carbonic anhydrase (CA) 1 and CA4. The increased bicarbonate concentration in cytosol activates RHC1, which suppresses HT1, a negative regulator for high CO_2_ induced stomatal closure. The inhibition of HT1 relieves OST1, which activates SLAC1 and facilitates stomatal closure by transporting Cl^−^ from cytosol to the intercellular space (Tian et al., 2015). OST1 also activates respiratory burst oxidase homolog (RBOH) D/F, which leads to ROS burst in the cytosol. Nitric oxide (NO) production is known to be downstream of ROS, and both H_2_O_2_ and NO can activate the tonoplast Ca^2+^ channel to trigger the release of Ca^2+^ into the cytosol. The increase in cytosol Ca^2+^ is an important step for CO_2_ induced stomatal closure (Webb et al., 1996; Young et al., 2006; Xue et al., 2011). A rapid type anion channel responsible for malate^2−^ export is also important in high CO_2_ induced stomatal closure (Meyer et al., 2010). Another malate transporter (AtABCB14), which mediates malate import from intercellular space, has been shown to be a negative regulator for high CO_2_ induced stomatal closure (Lee et al., 2008). In our previous study, we observed increases of jasmonic acids (JAs) in guard cells under high CO_2_. The increase was not observed in the *ca1ca4* mutant, JA biosynthesis and signaling mutants, which exhibited impaired high CO_2_ responses (Geng et al., 2016). These results indicate a potential role of JA in high CO_2_ induced stomatal closure.

Despite significant progress made in guard cell elevated CO_2_ response, the mechanisms for low CO_2_ induced stomatal opening remain elusive. Genetic studies have revealed that several *Arabidopsis* mutants (e.g., *ca1ca4, ht1, ost1, slac1*, and *rboh*) showed different levels of insensitivity to low CO_2_ induced stomatal opening and *ht1* completely lost the low CO_2_ response (Hashimoto et al., 2006; Matrosova et al., 2015). The double mutant *ost1ht1* has an intermediate level of stomatal conductance of *ost1* and *ht1* mutants, but does not have a low CO_2_ response, indicating HT1 is epistatic of OST1 in low CO_2_ induced stomatal opening (Matrosova et al., 2015).

Except for the physiological and genetics studies, little is known about the molecular mechanisms including metabolic regulations underlying the guard cell low CO_2_ responses. Small molecule metabolites are important regulators in stomatal movement with roles in osmotic regulation, signaling and redox regulation (Misra et al., 2015). For example, sugars, sugar alcohols and organic acids can contribute to the turgor pressure together with inorganic ions in course of stomatal movement. The most extensively studied organic osmolytes in guard cells are malate and sucrose (Lee et al., 2008; Lawson et al., 2014; Daloso et al., 2015a,b; Medeiros et al., 2016). Signaling molecules in guard cells include phytohormones (Daszkowska-Golec and Szarejko, 2013; Misra et al., 2015; Murata et al., 2015), lipids (Sakaki et al., 1995; Jung et al., 2002), zeaxanthin (Zeiger and Zhu, 1998; Zhu et al., 1998; Assmann, 1999), as well as cGMP (Pharmawati et al., 1998). The glutathione-ascorbate cycle is a well-known ROS scavenging pathway. In addition, flavonols have recently been shown to play a role in ROS scavenging, and can repress ABA induced stomatal closure (Watkins et al., 2014).

To discover new molecular components in low CO_2_-induced stomatal opening, we applied hyphenated mass spectrometry (MS)-based metabolomics and proteomics approaches to analyze short-term low CO_2_ responses in *B. napus* guard cells. The isolated guard cells were exposed to 400 ppm CO_2_ (control) and 0 ppm CO_2_ (treatment) and analyzed at five time points (0, 5, 10, 30, and 60 min). A total of 411 metabolites were quantified. We observed decreased trends of primary metabolites (e.g., most common amino acids, nucleotides and sugars), and changes in the levels of osmoregulators (e.g., increased levels of malate and mannitol at early and late time points). In contrast to guard cell elevated CO_2_ response (Geng et al., 2016), JA biosynthesis was not altered in the low CO_2_ response. Instead, phytohormones that induce stomatal opening, including cytokinins, auxins, gibberellins (GA), as well as melatonin increased at early time points. This study highlights the utility of single cell-type omics in discovering and testing new molecular components in cellular signaling and metabolic networks.

## Experimental procedures

### Plant materials

*B. napus* var. Global seeds obtained from Svalöv Weibull AB (Svalöv, Sweden) were grown as previously described (Zhu et al., 2010). Fully expanded leaves from well-watered 7 weeks old plants were used for stomatal movement analysis and guard cell purification. *A. thaliana* mutant seeds *coi1-12* were provided by Dr. Zhonglin Mou, Department of Microbiology and Cell Science, University of Florida, and *ca1ca4* and *lox2* mutant seeds were obtained from the Arabidopsis Biological Resource Center (ABRC, Ohio State University, Columbus, USA). Seeds were germinated on a half strength Murashige and Skoog (MS) (Murashige and Skoog, 1962) medium prior to transferring to a Metro-Mix 500 potting mixture (The Scotts Co., Marysville, OH, USA). Arabidopsis plants were grown under a photosynthetic flux of 140 μmol photons m^−2^ s^−1^ and an 8 h light (22°C)/16 h dark (18°C) cycle for 6 weeks.

### Preparation of epidermal peels for stomatal movement assay

Small squares (0.5 × 0.5 cm^2^) of leaf sections from 7 week-old *B. napus* plants were fixed abaxial side down onto coverslips coated with medical adhesive (Hollister, Libertyville, IL, USA). Adaxial epidermis and mesophyll layers were removed with a scalpel. After washing three times with distilled water to remove cellular debris, the coverslips were incubated with a cell wall digesting enzyme mixture at 26°C, 140 excursions per min for 13 min on a reciprocal shaker. The digesting enzyme mixture was constituted as follows: 0.7% cellulase R-10 (Yakult Honsha Co., Ltd, Tokyo, Japan), 0.025% macerozyme R-10 (Yakult Honsha Co., Ltd, Tokyo, Japan), 0.1% (w/v) polyvinylpyrrolidone-40 (Calbiochem, Billerica, Massachusetts, USA), and 0.25% (w/v) bovine serum albumin (Research Products International Corp., Mt Prospect, Illinois, USA) in 55% basic solution (0.55 M sorbitol, 0.5 mM CaCl_2_, 0.5 mM MgCl_2_, 0.5 mM ascorbic acid, 10 μM KH_2_PO_4_, 5 mM 4-morpholineethanesulfonic acid (MES), pH 5.5 adjusted with 1 M KOH). The coverslips were then incubated in an opening buffer (10 mM KCl, 50 μM CaCl_2_, 10 mM MES-KOH, adjusted to pH 6.15 with 1 M KOH) under light (110 μmol m^−2^ s^−1^) for an hour. For low CO_2_ treatment, the stomata were first stabilized in 400 ppm CO_2_ balanced opening buffer for 15 min and then exposed continuously to 0 ppm CO_2_ balanced opening buffer for a period of 60 min as previously described (Hu et al., 2010; Geng et al., 2016).

### Large scale enriched stomata preparation for proteomics and metabolomics analysis

Stomata from *B. napus* were prepared as previously described (Zhu et al., 2012; Geng et al., 2016). Briefly, the main and secondary veins of leaves were removed with a scalpel. Then the leaves were blended for 20 s each time for a total of four times in a blender (Sunbeam Products, Inc., Boca Raton, FL, USA) to remove the mesophyll cells. Epidermal peels were collected on a 100 μm nylon mesh and washed thoroughly with tap water to remove any broken cells. The peels were transferred to the enzyme mixture and digested as described above. Enriched stomata on digested peels were collected on the 100 μm nylon mesh and washed thoroughly with the opening buffer three times, and then transferred to the opening buffer in square petri dishes for recovery under light (140 μmol photons m^−2^ s^−1^) at 22°C for an hour. All the samples were stabilized in 400 ppm CO_2_ (control) for 15 min before continuing in control or switching to 0 ppm CO_2_ treatment (Hu et al., 2010). The samples were collected at 0, 5, 10, 30, and 60 min on nylon meshes of 100 μm pore size, blotted dry quickly on filter paper, and transferred to Eppendorf tubes, and then quickly frozen (~30 s) in liquid nitrogen and stored at −80°C before protein extraction. The samples for metabolomics were lyophilized to dryness before metabolite extraction.

### Metabolite extraction and sample preparation for metabolomics

Each independent stomatal preparation was regarded as a replicate. A total of four replicates, each of 100 mg lyophilized sample were sent to Metabolon® Inc. (Durham, NC, USA) for GC-MS and UPLC-MS based metabolomic analyses. The samples were extracted sequentially with four different extraction solvents, i.e., 400 μL tridecanoic acid (2.5 mg/mL) dissolved in ethyl acetate:ethyl alcohol (1:1), 200 μL methanol, 200 μL methanol:H_2_O (3:1), and 200 μL dichloromethane:methanol (1:1). Each extraction was carried out at 4°C on a Geno/Grinder 2000 homogenizer (with a 4 mm stainless steel ball added to each tube) (Glen Mills Inc., NJ, USA) for 2 min in the above specified extraction buffers, and the supernatants of each extraction were collected after centrifugation and pooled. Pooled extracts were divided into four equal aliquots, one used for GC-MS and three used for UPLC-MS. For GC-MS, the aliquots were lyophilized (Centrivac, Labconco Inc.) and further derivatized using 1:1 mixture of bistrimethylsilyl-trifluoroacetamide (Sigma, St. Louis, USA) and acetonitrile:dichloromethane:cyclohexane (5:4:1 by volume) with 5% trimethylamine and a series of alkylbenzenes used as retention index (RI) markers (Roessner-Tunali et al., 2003) at 60°C for 1 h. For UPLC-MS, the samples were also lyophilized and reconstituted in different loading solvents (water with 0.1% formic acid for reverse phase positive mode, 0.1% formic acid in 6.5 mM ammonium bicarbonate for reverse phase negative mode, and 10 mM ammonium formate for HILIC negative mode). The RI standards for the reverse phase positive mode include d7-glucose (RI 702), fluorophenylglycine (RI 1185), d3-methionine (RI 1237), d4-tyrosine (RI 1506), d3-leucine (RI 1666), d8-phenylalanine (RI 2027), d5-tryptophan (RI 2422), d5-hippuric acid (RI 2772), Br-phenylalanine (RI 3168), d5-indole acetate (RI 3740), d9-progeterone (RI 5298) and d4-diocylphthalate (RI 6118). Those for the negative mode include d7-glucose (RI 706), d3-methionine (RI 1134), d3-leucine (RI 1467), d8-phenylalanine (RI 1992), d5-tryptophan (RI 2264), Br-phenylalanine (RI 3058), Cl-phenylalanine (RI 3308), d15-octanoic acid (RI 4317), d19-decanoic acid (RI 5073), d27-tetradecanoic acid (RI 5423) and d35-octadecanoic acid (RI 5833).

Another set of four replicates of lyophilized samples (prepared separately) was used for our in-house targeted multiple reaction monitoring (MRM) HPLC-MS analyses (Supplementary Table S1) as previously described (Geng et al., 2016). Briefly, 10 μL internal standard mixture (100 μM lidocaine and 100 μM 10-camphorsulfonic acid for evaluating extraction efficiency) was added to each sample, and the samples were homogenized (with a 4 mm stainless steel ball in each tube) in liquid nitrogen for 20 s at 1900 strokes/min in a GenoGrinder (Geno/Grinder 2000, SPEX SamplePrep., Metuchen, NJ, USA). Metabolites were sequentially extracted in 1 mL of each extraction solvent, i.e., 80% methanol, acetonitrile:isopropanol:water (3:3:2), and acetonitrile:water (1:1) on a thermomixer (Thermomixer R, Eppendorf, Hamburg, Germany) at 4°C, 1,100 rpm for 15 min. Samples were then sonicated in ice water using a Branson® Ultrasonic Bath at 50 Hz for 15 min and centrifuged at 13,000 g for 15 min at 4°C. The combined supernatants were lyophilized at 160 mBar (Centrivap, Labconco Inc., USA) and re-dissolved in 100 μL water at room temperature for 15 min. After centrifugation at 4°C, 13,000 g for 15 min, the supernatants were used for MRM HPLC-MS.

### GC-MS, UPLC-MS and MRM HPLC-MS analyses

Samples for GC-MS and UPLC-MS analyses were profiled as previously described (Lawton et al., 2008; Evans et al., 2014). Briefly, derivatized GC-MS samples were separated on a 5% diphenyl/95% dimethyl polysiloxane fused silica column (20 m × 0.18 mm ID; 0.18 um film thickness, Thermo Scientific Inc.) with helium as carrier gas and a temperature ramp from 60 to 340°C in 17.5 min, followed by detection on a Trace DSQ quadruple mass spectrometer with electron impact ionization (Thermo Scientific Inc., San Jose, CA, USA). For UPLC-MS, the columns were a Waters UPLC BEH C18 (2.1 × 100 mm, 1.7 μm) column for reverse phase chromatography, and a Waters UPLC BEH Amide (2.1 × 150 mm, 1.7 μm) column for the hydrophilic interaction liquid chromatography (HILIC) separation. The solvents used for reverse phase positive mode were 0.1% formic acid in water (solvent A) and methanol (solvent B), for reverse phase negative mode is 6.5 mM ammonium bicarbonate in water (A) and methanol (B), and for HILIC negative mode is 10 mM ammonium formate in water (A) and acetonitrile (B). The gradient of solvent B for reverse phase was ramped from 0.5 to 70% in 4 min, then from 70 to 98% in 0.5 min, held at 98% for 0.9 min, then return to 0.5% in 0.2 min, and finally held at 0.5% for 5.4 min. The flow rate was 0.35 mL/min. The gradient for HILIC was ramped from 5 to 50% in 3.5 min, then to 95% in 2 min, held at 95% for 1 min, and back to 5% in 0.2 min and held at 5% for 4.3 min. The flow rate was 0.5 mL/min. The Waters ACQUITY UPLC was connected to a Thermo Scientific Q-Exactive mass spectrometer interfaced with a heated electrospray ionization (HESI-II) source. The MS analysis alternated between MS and data-dependent MS2 scans using dynamic exclusion, and the scan range was from 80 to 1,000 m/z. The spray voltage for reverse phase positive mode, negative mode and HILIC negative mode was 4, 2.75, and 3 kV, respectively. The sheath gas and auxiliary gas settings for reverse phase positive mode were 40 arbitrary units (au) and 5 au, respectively. Those for reverse phase negative mode were 75 au and 15 au, respectively, and for HILIC were 40 au and 5 au, respectively. The source heater temperature for reverse phase positive and negative mode was 400°C, and for HILIC was 380°C. The normalized collision energy setting for reverse phase positive mode, negative mode and HILIC negative mode was 40, 60, and 60%, respectively.

HPLC-MRM-MS was conducted using an Agilent 1100 HPLC (Agilent, Santa Clara, CA, USA) coupled with an AB Sciex 4000 QTRAP™ (AB Sciex, Framingham, MA, USA). An Agilent, Eclipse XDB-C18 column (4.6 × 250 mm, 5 μm) was used for metabolite separation with 0.1% formic acid in water as solvent A and 0.1% formic acid in acetonitrile as solvent B. The HPLC gradient started at 1% solvent B for 5 min, then a linear gradient from 1 B to 99.5% B over 41.5 min, held at 99.5% B for 4.5 min, and then return to 1% B in 0.3 min and held at 1% B for 8.7 min. The flow rate was 0.5 mL/min. The mass spectrometer conditions were: 30 psi curtain gas, 50 psi GS1, 55 psi GS2, ion source voltage 4,500 V, with a TurboIon electrospray ionization (ESI) interface temperature of 350°C. A multiple period method was used as previously described (Chen et al., 2013; Geng et al., 2016). Briefly, MRM-transitions were separated into different periods based on the compounds retention time to increase the number of compounds that can be detected in a single run. The five periods in positive mode were separated at 5.8, 15.7, 23.7, 36.2, and 60 min. The three periods in negative mode were separated at 15.9, 26.32, and 60 min. Both positive and negative modes use the same HPLC gradient as described above. Detailed compounds in each period of the MRM method can be found in Supplementary Table S1.

### Metabolomics data processing, statistics and pathway analysis

All data were combined to a final peak list (Supplementary Table S1). The data processing method was described in detail in previous publications (Evans et al., 2009, 2014). Briefly, a Metabolon in-house software was used for metabolite peak detection and integration. Extracted ion chromatograms were binned by mass. After determining the baseline noise, MS peaks were determined and peak areas were calculated based on these criteria: smoothing of 7–9, signal to noise >5, height threshold of 20,000, width threshold of 0.035, area threshold of 20,000, and 10% zero scans (essentially permitted number of missing scans allowed for peak to be considered real). Individual MS peaks were grouped by apex retention time, and the chromatograms were aligned based on RI values. The RI of a compound is a number, obtained by logarithmic interpolation, relating the adjusted retention volume (time) or the retention factor of the compound to the adjusted retention volumes (times) of two RI standards (whose RI values were set) eluted before and after the peak of the sample component (Kováts, 1958; Evans et al., 2009). The output data after peak detection and integration contained m/z ratios, RIs and peak areas.

Metabolite identification was relied on comparing RI, accurate mass +/− 0.005 amu and MS/MS spectral data with the authentic compound information in the Metabolon library. The RI should be within 75 RI units of the proposed identification (Evans et al., 2009). The MS/MS matching is based on a comparison of the ions present in the experimental spectrum to the ions present in the library spectrum. All proposed identifications were manually reviewed and hand curated based on the above mentioned criteria. For metabolites identified from multiple platforms (e.g., GC-MS and LC-MS), only one call of highest quality for a given compound (with symmetry narrow peak and a tailing factor close to 1, as well as lack of interference from adjacent compounds based on isotopic patterns) was chosen so that the statistics will not be skewed. If one platform had missing data (nulls) and another had values for the samples, the latter was chosen. The peak area (raw) for one ion in each compound spectrum was used for quantification. The data have no units, and represent relative quantification. Comparisons of peak values are valid only within each compound individually, across all samples. Missing values were imputed using a k-Nearest Neighbor (KNN) method (Hastie et al., 2014). Differentially changed metabolites were analyzed using R-based empirical analysis of digital gene expression (EDGE) package in R (version 3.2.5) (R Development Core Team, 2015) as previously described (Jin et al., 2013; Storey et al., 2015). Comparisons were made between treatment and control samples for each time point. The full model used CO_2_ concentration as the variable of interest, replicate batch as adjustment variables, and a null model used only replicate batch as adjustment variables. The R package uses likelihood ratio test to generate *p*-values. A total of 2,000 bootstrap iterations were performed for each comparison. Metabolic pathway analyses were performed for metabolite comparisons at each time point using MetaboAnalyst 3.0 (Xia et al., 2015). Raw data were uploaded to MetaboAnalyst 3.0 webserver and only metabolites mapped to the KEGG pathway database were used. A log_2_ transformation was used to normalized and scale the data before further analysis.

### Protein extraction and tandem mass tag (TMT) 10-plex labeling

Enriched stomata samples (each of 100 mg fresh weight) were ground with a mortar and a pestle in liquid nitrogen into fine powder, which was transferred to a 40 mL Oakridge tube. Then 1.25 mL of 10 mM Tris-HCl, pH 8.0 saturated phenol and 1.25 mL of extraction buffer (0.1 M Tris-HCl pH8.8, 10 mM EDTA, 0.4% β-mercaptoethanol and 0.9 M sucrose) were added to the sample sequentially. The mixture was agitated at room temperature for 2 h, followed by centrifugation at 4°C, 5,000 g for 10 min. The phenol layer was transferred to a new tube, and another 1.25 mL phenol was added to the bottom layer to back extract at room temperature for 30 min. The phenol layer was combined with the previous extraction and protein was precipitated with 5 volumes of cold 0.1 M ammonium acetate in 100% methanol at −20°C overnight. The protein was then pelleted by centrifugation at 4°C, 20,000 g for 20 min. The pellet was washed twice with 0.1 M ammonium acetate, twice with 80% acetone and once with 100% acetone. Protein amounts were assayed using a EZQ assay kit (Thermo Scientific Inc.) according to manufacture instructions. A total of 30 μg protein each sample was dissolved in 50 μl 100 mM triethyl ammonium bicarbonate (TEAB) buffer, followed by reduction with 17 mM Tris (2-carboxyethyl) phosphine at room temperature for 30 min and alkylation with 10 mM iodoacetamide for 1 h in the dark. Six volumes of pre-chilled (−20°C) acetone was used to precipitate the protein at −20°C overnight. Samples were then centrifuged at 4°C at 8,000 g for 10 min. Supernatant was carefully removed and pellets were left to dry for 2–3 min. Then, 50 μL of 100 mM TEAB was used to re-dissolve the sample and 1.5 μg of trypsin was used to digest the protein at 37°C overnight. TMT labels 126, 127N, 127C, 128N, 128C, 129N, 129C, 130N, 130C, and 131 were used to label samples collected at 0 min, 5 min control, 5 min treatment, 10 min control, 10 min treatment, 30 min control, 30 min treatment, 60 min control, 60 min treatment, and a universal control sample, respectively. Samples were labeled for 1 h and then quenched with 4 μL of 5% hydroxylamine for 15 min. All samples were then combined, lyophilized, and reconstituted in 100 μL 0.1% TFA (trifluoroacetic acid) solution. A Macrospin C-18 reverse phase mini-column with a capacity of 30–300 μg (The Nestgroup Inc., Southborough, MA, USA) was used to desalt the samples. Two independent replicate TMT experiments were conducted.

### Strong cation exchange fractionation and UPLC-MS/MS analysis of peptides

The TMT labeled peptides were fractionated using an Agilent HPLC 1260 on a polysulfoethyl A strong cation exchange (SCX) column (2.1 × 100 mm, 5 μm, 300 Å, PolyLC, Columbia, USA). Solvent A was 25% (v/v) acetonitrile, 10mM ammonium formate and 0.1% (v/v) formic acid (pH 2.8), and solvent B was 25% (v/v) acetonitrile and 500 mM ammonium formate (pH 6.8). The gradient for solvent B was at 0% for 10 min, then ramped up to 20% in 80 min and to 100% in 5 min, held at 100% for 10 min. Peptide elution was monitored at 280 nm and 12 fractions were collected for UPLC-MS/MS.

The SCX fractions were analyzed on an Easy-nLC 1200 system coupled to a Q-Exactive Orbitrap Plus MS (Thermo Fisher Scientific, Bremen, Germany). The peptides were concentrated on an Acclaim PepMap 100 pre-column (20 mm × 75 μm; 3 μm-C_18_), then separated on a PepMap RSLC analytical column (250 mm × 75 μm; 2 μm-C_18_). Water and 80% acetonitrile with 0.1% formic acid were used as solvent A and B, respectively. The gradient for solvent B ramped from 2 to 30% in 100 min, from 30 to 98% in 10 min, then held at 98% for 10 min. The flow rate was 350 nL/min. Positive ion mode with data dependent scanning and higher-energy collision dissociation (HCD) was used as previously described (Jones et al., 2013). The full MS resolution was 70,000 with a scan range of 400–2,000 m/z, an automatic gain control (AGC) target of 1e^6^ and maximum ion trapping (IT) of 100 ms. The tandem mass spectrum resolution was 35,000, an AGC target of 2e^5^, maximum injection time of 117 ms, a loop count of 10, an isolation window of 1.3 m/z, a fixed first mass of 115 m/z and a normalized collision energy setting of 32%. A lock mass of polysiloxane ion (445.12,003 m/z) was used for real-time mass calibration. The spray voltage was set to be 1900 volts, capillary temperature was 320°C, and the stacked ring ion guide radio frequency voltage was set to 70 V.

### Protein identification and quantification

Peptides were identified using Proteome Discoverer 1.4 software (Thermo Fisher Scientific, Bremen, Germany), searched against a modified *B. napus* database downloaded from Genescope (Chalhoub et al., 2014). Redundant sequences were first removed with patpd program from AB-BLAST 3.0 (Gish, 1996-2009), then subjected to redundant entry clustering using the perl script nrdb90 with an identity level at 95% (Holm and Sander, 1998). The final database contains 79,366 non-redundant proteins. The annotation was done by blasting against the non-redundant green plant database from NCBI using Blast2Go (Conesa and Götz, 2008). The searching parameters include 20 ppm tolerance for precursor mass tolerance, and 0.02 Dalton for fragment mass tolerance, two missed cleavages, TMT10-plex label on the N-terminal, carbamidomethylation (C), oxidation (M), phosphorylation (S, T, Y), and deamidation on the N-termini. Peptides were filtered using stringent Xcorr value cut off at 2.31 for 2+, 2.41 for 3+, 2.6 for 4+ and 5+ peptides. Peptide quantification result was exported for further processing and statistical analysis. The peptide quantification was first normalized by the mean of each tag, then sum of the same peptide was calculated. Only peptides that were present in the two independent experiments and unique for their corresponding proteins were used for protein quantification. All the identified peptides and proteins are listed in Supplementary Table S2. Ratios of 0 ppm CO_2_ treated samples to 400 ppm CO_2_ treated controls were calculated for each time point (127C/127N for 5 min, 128C/128N for 10 min, 129C/129N for 30 min, 130C/120N for 60 min). Fold changes for proteins were the means of all unique peptides identified from the corresponding proteins. Student's *t*-tests were performed with the log_2_ transformed fold changes for each time point. A singular enrichment analysis (SEA) was performed for proteins with a *p*-value less than 0.05 using agriGO (Du et al., 2010).

## Results

### Low CO_2_ induced stomatal opening and metabolomic changes

**Figure 1:**
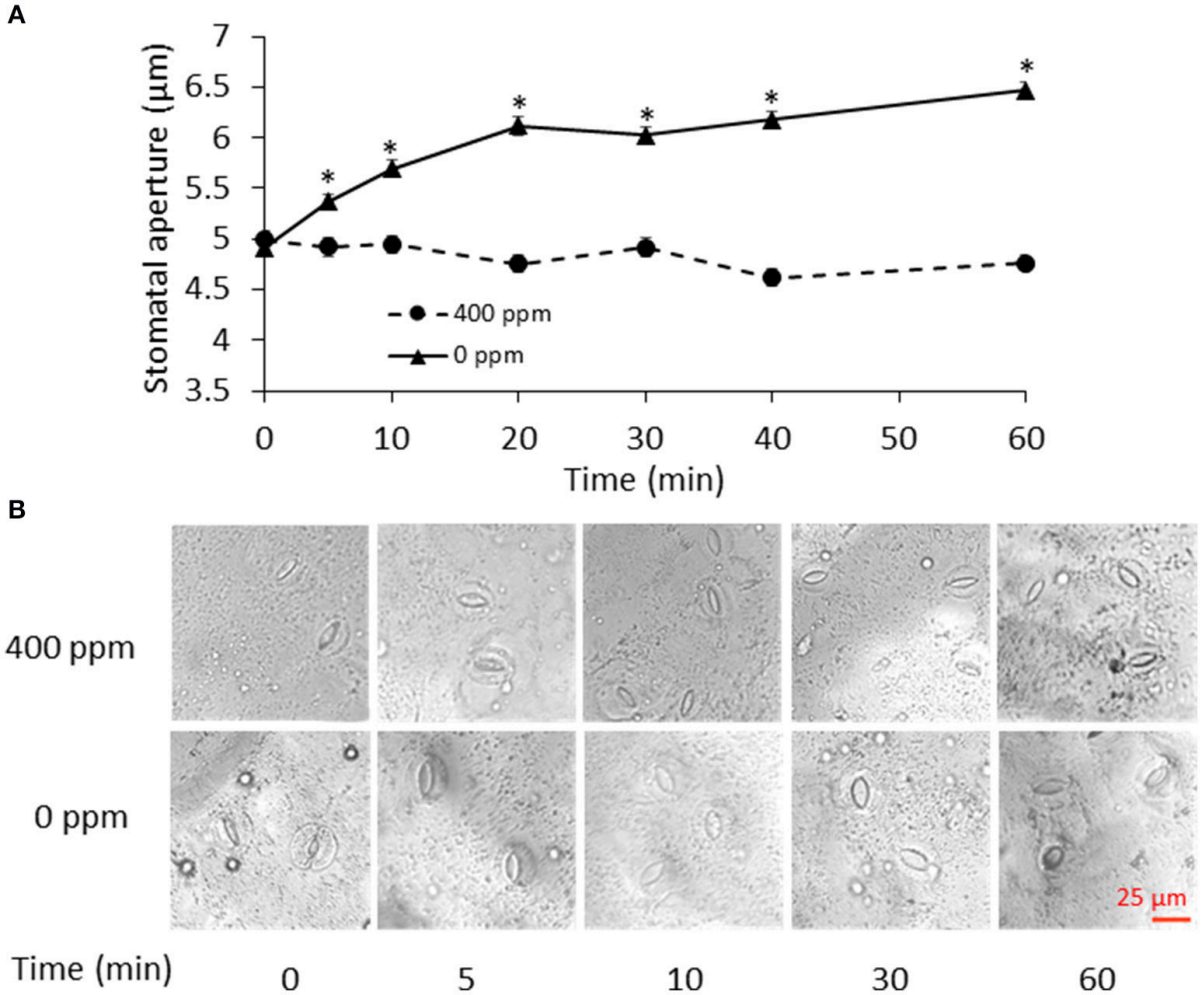
Stomatal movement in response to low CO_2_. **(A)** Time course of stomatal movement under atmospheric (400 ppm) and low CO_2_ (0 ppm) conditions. Data are mean ± standard error of three independent experiments (*n* = 3) with 60 stomata counted for each replicate. ^*^p < 0.05, Student's *t*-test. **(B)** Representative images of stomatal aperture during the control atmospheric CO_2_ and low CO_2_ treatments. Scale bar indicates 25 μm.

To determine the time points for metabolite measurement, *B. napus* stomatal movements in response to ambient (400 ppm) and low (0 ppm) CO_2_ conditions were recorded at different time points. Stomatal aperture under low CO_2_ treatment gradually increased, and became significantly different from the ambient control samples at 5 min and onwards (Figure 1). The fast response to low CO_2_ took place within 20 min. At 60 min, there was only a slight increase in stomatal aperture relative to the previous three time-points (Figure 1). Based on this data, we chose to collect our guard cell samples at 0, 5, 10, 30, and 60 min after the low CO_2_ treatment.

**Figure 2:**
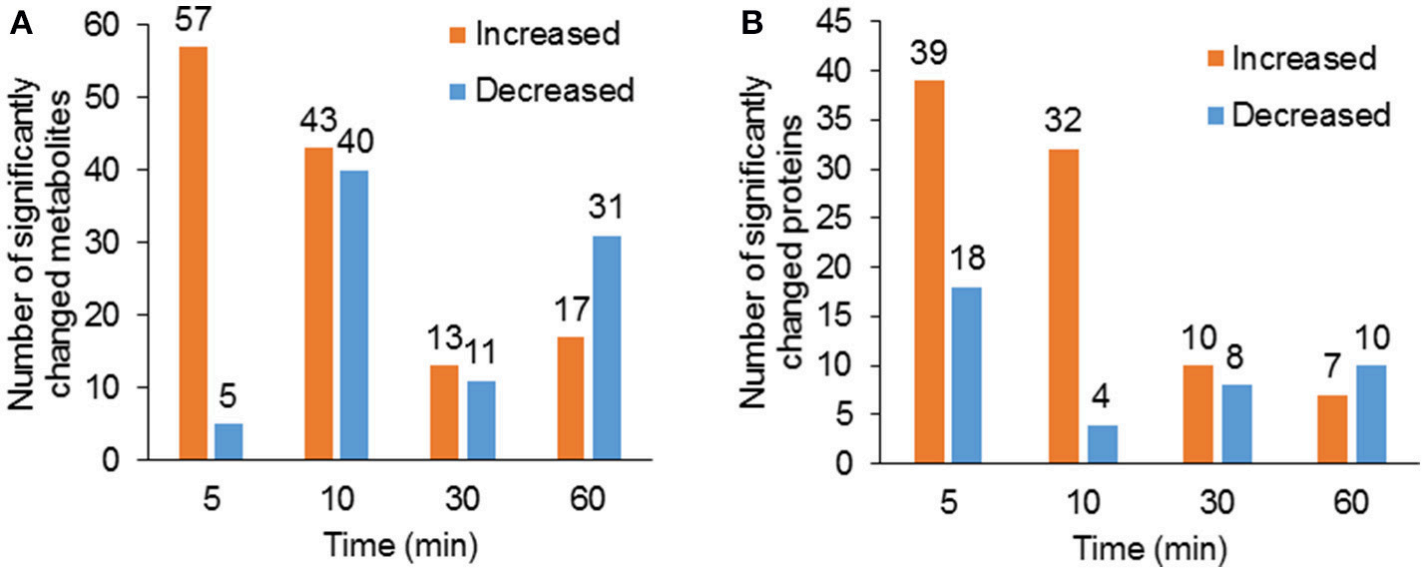
Overview of significantly changed metabolites and proteins under low CO_2_ condition. **(A)** Total number of significantly increased (*p* < 0.05, fold change >1.2) and decreased (*p* < 0.05, fold change <0.8) metabolites at each time point of the treatment. **(B)** Total number of significantly increased and decreased proteins at each time point of the treatment.

**Figure 3:**
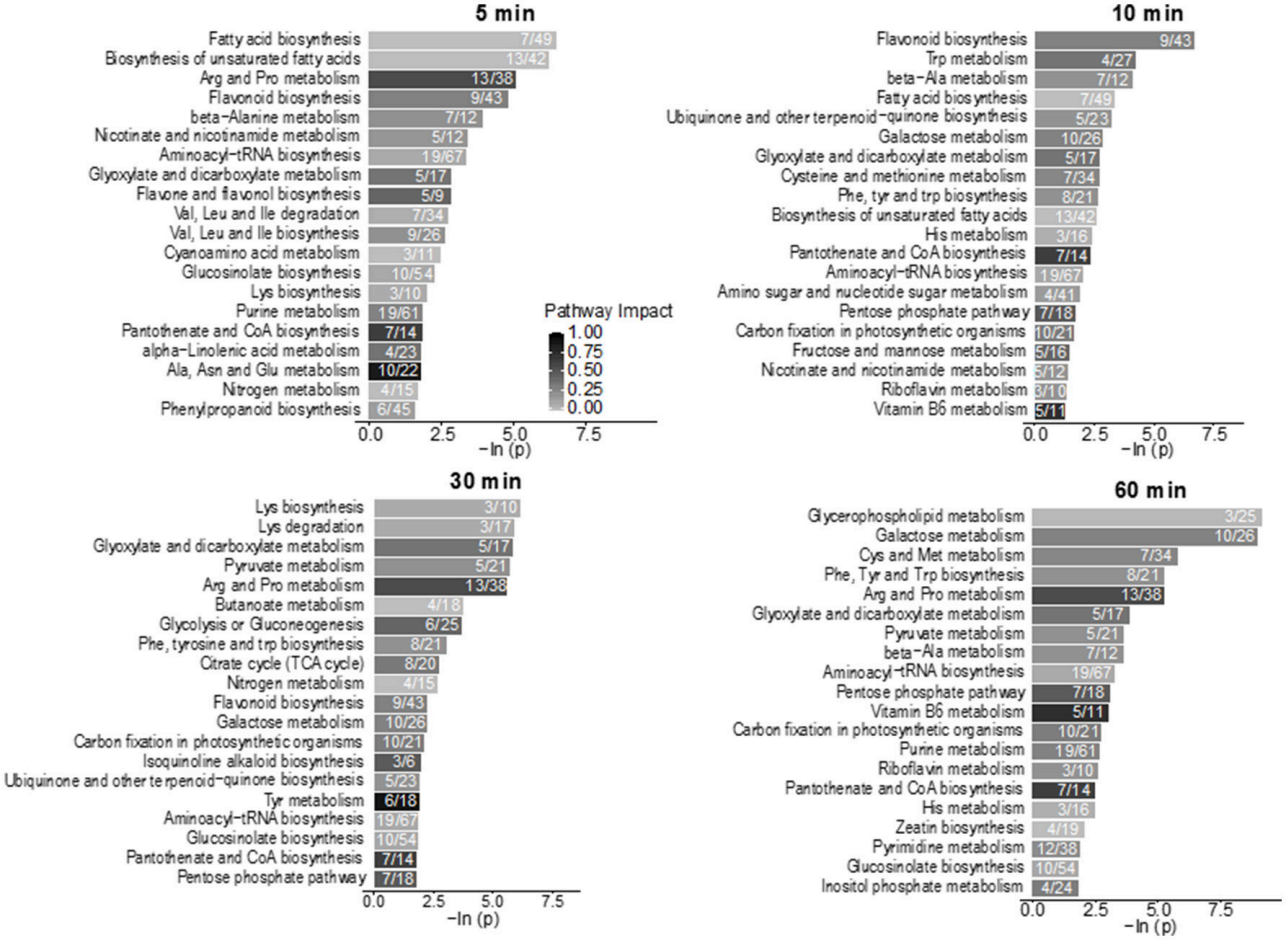
Pathway enrichment analysis of the low CO_2_ responsive metabolites in the time-course study. Numbers inside the bars indicate the number of metabolites that matched to the total metabolites in that pathway. Only pathways with four or more metabolites that matched were included in the analysis. The scale bar of different levels of grayness indicates pathway impact.

**Figure 4:**
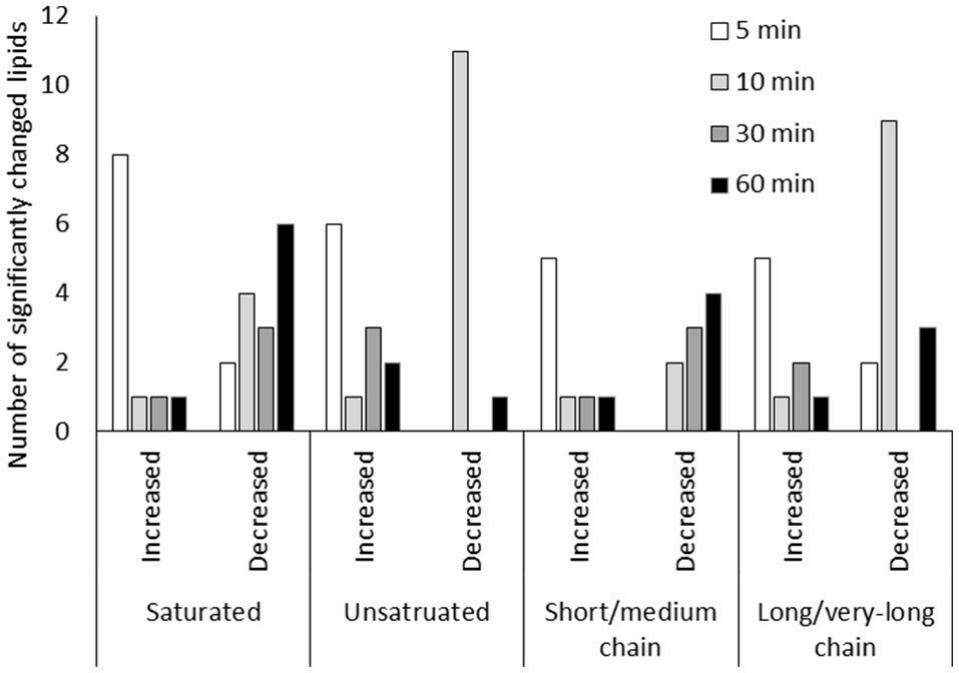
Lipid metabolic changes in guard cells under low CO_2_. Short/medium chain lipids include lipids of 5C-14C lipids. Long/very-long chain lipids include lipids with carbon chains longer than 16C. Only lipids that showed significant changes (*p* < 0.05) were included.

Using hyphenated-MS based metabolomics platforms, we identified 411 non-redundant metabolites in stomatal guard cells. The numbers of differentially changed metabolites at each time point were shown in Figure 2A. Before 30 min of low CO_2_ treatment, there were more increased metabolites than decreased ones. At 60 min, the decreased metabolites outnumbered the increased metabolites (Figure 2A). The initial increase in many metabolites could be attributed to catabolism from large storage molecules and production of osmolytes needed for turgor and stomatal opening. At 60 min, more decreased metabolites may reflect decreased inorganic carbon source under the low CO_2_ condition. The significantly accumulated metabolites are specific to each time point and belong to different metabolic pathways. At 5 min, pathway enrichment analysis revealed that intermediates in unsaturated fatty acid biosynthesis were significantly over-represented among the significantly altered metabolite levels. At 5 min and 10 min, fatty acid and flavonoid biosynthesis were over-represented (Figures 3, 4), and these two pathways were also found to be responsive to elevated CO_2_ (Geng et al., 2016).

### Lipid metabolism in guard cells under low CO_2_

Lipid metabolism showed significant changes at 5 min and 10 min based on the pathway enrichment result (Figure 3). Since many of the lipids identified cannot be assigned with a Kyoto Encyclopedia of Genes and Genomes (KEGG) ID, here we manually examined the lipid species detected, which were mainly short chain free fatty acids and monoacylglycerol (Supplementary Table S1). At 5 min, most lipids and fatty acids increased, but after 10 min most lipids decreased (Figure 4). This trend of change was similar for both saturated and unsaturated lipids, irrespective of their different chain lengths. Free fatty acid accumulation during the low CO_2_ induced stomatal opening could be caused by increased hydrolysis of intracellular lipids, increased de-novo synthesis or decreased consumption by free fatty acid-consuming pathways. The mechanisms causing subsequent decreases in free fatty acids after 10 min are unknown, but could be explained by their incorporation into membranes or catabolism for energy production. Interestingly, our proteomics data showed that under low CO_2_, proteins involved in catabolism process are enriched (Supplementary Figure S1). Thus, the initial increase in fatty acids is likely due to the breakdown of storage lipids. Concomitantly, at 5 min two GDSL (Glutamate-Aspartate-Serine-Leucine) motif containing esterases/lipases were found to be significantly increased (Supplementary Table S3) and these enzymes usually exhibit broad substrate specificity and multi-functionality (Chepyshko et al., 2012). Whether they directly hydrolyze storage lipids is not known.

At 5 min, two phospholipids (Supplementary Table S1) and a phospholipase Dα (Supplementary Table S3) decreased. The product of phospholipase Dα has been shown to be a positive regulator of ABA induced stomatal closure (Zhang et al., 2009). Thus, the decreased levels of phospholipids and phospholipase are consistent with the stomatal opening phenotype under low CO_2_ (Figure 1).

### Phytohormone changes in guard cells under low CO_2_

**Figure 5:**
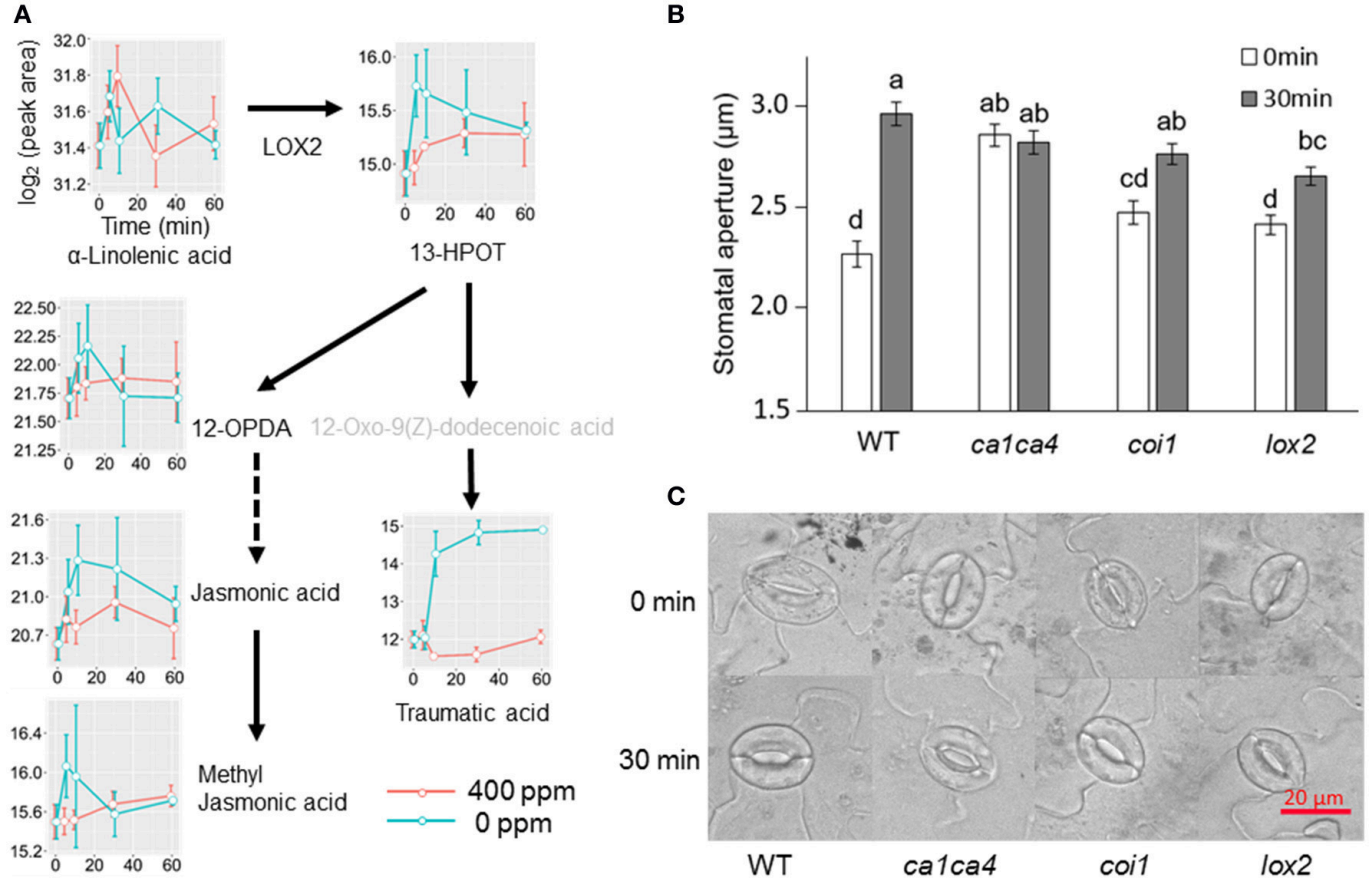
Metabolite changes in JA and traumatic acid pathways and stomatal low CO_2_ response of *A. thaliana* mutants. **(A)** Changes of metabolites in JA and traumatic acid pathways in *B. napus* guard cells under low CO_2_ treatment. 13-HPOT, (9Z,11E,15Z)-(13S)-13-Hydroperoxyoctadeca-9,11,15-trienoic acid; 12-OPDA, (15Z)-12-Oxophyto-10,15-dienoic acid; **(B)** Stomatal movement of JA biosynthesis and signaling mutants after low CO_2_ treatment. Data are mean ± standard error of three independent experiments (*n* = 3) with 60 stomata counted for each replicate (i.e., a total of 180 stomata). Two-way ANOVA and Tukey's test were used for stomatal movement analysis between different time points and different genotypes; **(C)** Representative images of stomatal aperture of **(B)**.

**Figure 6:**
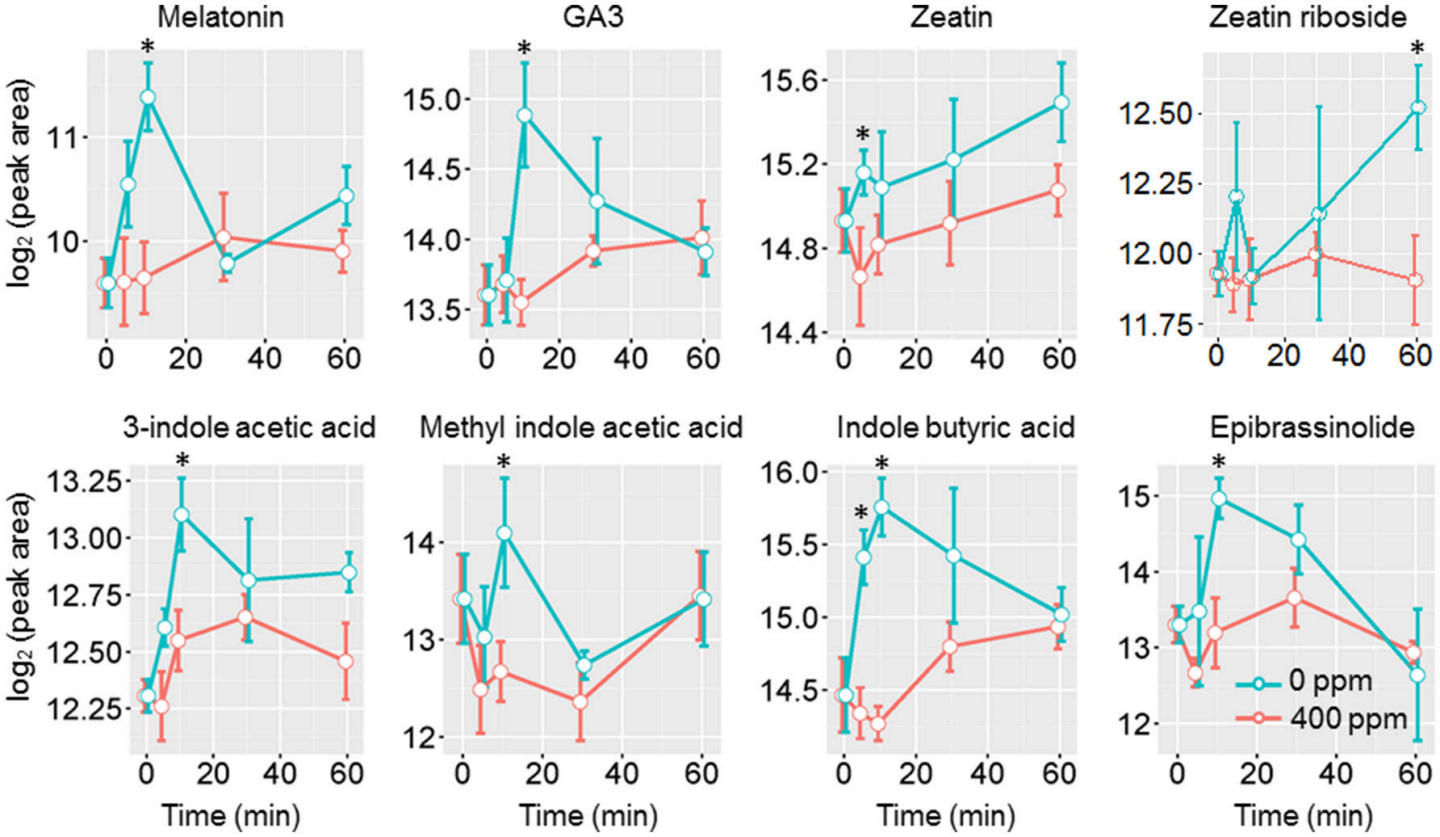
Phytohormone level changes in guard cells under low CO_2_. Student's *t*-test was performed between atmospheric control and low CO_2_ treatment at each time point. Star (^*^) indicates *p* < 0.05.

Jasmonic acids were previously shown to be involved in high CO_2_ induced stomatal closure (Geng et al., 2016). Under low CO_2_, however, we only observed an increase of the intermediate 13S-hydroperoxy-9Z,11E,15Z-octadecatrienoic acid (13-HPOT), which is a precursor for jasmonates (JAs) and traumatic acid production. No significant changes were found in jasmonic acid, methyljasmonic acid or 12-oxo-phytodienoic acid. Interestingly, traumatic acid showed more than 7-fold increases after 10 min of low CO_2_ treatment (Figure 5). That traumatic acid and not jasmonic acid was accumulated indicates that traumatin may be more important than JAs in low CO_2_ induced stomatal opening. We then tested the stomatal movement of JA biosynthesis mutant *lox2* and signaling mutant *coi1*. Both mutants were still able to open stomata in response to low CO_2_ (Figure 5), confirming that JAs are not involved in guard cell low CO_2_ response. Similar to JAs, abscisic acid (ABA) did not show significant changes under low CO_2_, but ABA catabolic products, phaseic acid (1.49-fold at 5 min) and dihydrophaseic acid (>2.4-folds after 10 min) showed significant increases (Supplementary Table S1). As expected, hormones known to induce stomatal opening, e.g., auxins, cytokinins, brassinosteroids, gibberellins all showed increasing trends under low CO_2_ (Figure 6). In addition, melatonin, a well-studied animal hormone regulating circadian rhythms and various biological processes and discovered in plants in 1995 (Arnao and Hernandez-Ruiz, 2006; Nawaz et al., 2016, was found to increase at 5 min and 10 min of low CO_2_ treatment (Figure 6). Except for zeatin riboside, all other hormones increased at early time points at 5 or 10 min. These data suggest that low CO_2_ induced stomatal opening is a multi-hormone response.

### Protein changes in guard cells under low CO_2_

To complement the metabolomics data, we performed a proteomic analysis of the samples used for metabolomics. The number of significantly changed proteins showed similar trends of changes as metabolites (Figure 2B). Single enrichment analysis was performed for proteins that show significant changes at one or more time points. The differentially accumulated proteins were enriched in metabolic processes, especially oxidation/reduction, catabolism/ proteolysis, and cellular nitrogen metabolism processes. In terms of molecular functions, these proteins were enriched in catalytic activity, protein and nucleotide binding, and antioxidant activity (Supplementary Figure S1). Here we discuss some examples (please refer to Supplementary Table S3 for detailed results of significant protein changes).

At 5 min a V-type ATPase subunit G1 was significantly increased (1.3-fold change, with a *p*-value of 0.03), and so was another subunit A2 at 10 min (1.4-fold, with a *p*-value of 0.04). The increase of V-type ATPases may cause cytosolic acidification under low CO_2_ and promote stomatal opening. Guard cell V-type ATPase activity was found to be insensitive to ABA or fusicoccin (Willmer et al., 1995), so it is possible that the V-type ATPase represents specific low CO_2_ response. At 5 min, a probable aquaporin PIP2-5 was increased (1.4-fold, with a *p*-value of 0.04). Aquaporin is one of the passages for CO_2_ intake. Although a knockout mutant *pip2;1* did not show any significant changes compared to wildtype in the course of high CO_2_ induced stomatal closure, PIP2-1 has been implicated in CO_2_ signal transduction since it physically interacts with CA4 located on the plasma membrane (Wang et al., 2016). How PIP2-5 functions in guard cell low CO_2_ response is intriguing. At 10 min, a voltage dependent potassium channel increased by 1.2-fold. The increases of the above mentioned membrane transport-related proteins may play important roles in promoting stomatal opening under low CO_2_. In addition, a subtilisin-like protease 2 was found to be increased at 5 min (1.5-fold, with a *p*-value of 0.03). Subtilisin-like protease SSD1 was shown to cleave EPIDERMAL PATTERNING FACTOR 2 (EPF2), producing the active form of EPF2. Loss of SSD1 was reported to enhance the elevated CO_2_ induced decrease of stomatal index (Engineer et al., 2014). It is reasonable to predict that increased SSD protein level may increase stomatal index under long-term low CO_2_ treatment.

Consistent with the phytohormone changes described in the previous section, auxin transporter 3 and gibberellin-regulated 14 like protein were found to increase significantly at 5 min (1.4-fold, with a *p*-value of 0.03) and 10 min (1.3-fold, with a *p*-value of 0.04), respectively. Another interesting change is the significant decrease of an isoflavone reductase homolog P3-like at 30 min after low CO_2_ treatment (0.76-fold, with a *p*-value of 0.01). Isoflavone reductase was reported to function as an antioxidant in response to ROS (Kim et al., 2010; Watkins et al., 2014). ROS burst is known to play an important signaling role in the regulation of stomatal closing processes (Watkins et al., 2014; Murata et al., 2015; Geng et al., 2016; Sierla et al., 2016), but it has not been observed in the stomatal opening process. The decrease of isoflavone reductase constitutes another line of evidence for the lack of ROS burst in the low CO_2_ induced stomatal opening.

## Discussion

### Fatty acid and energy metabolism are important in stomatal low CO_2_ response

In general, primary metabolism in guard cells under low CO_2_ is skewed toward catabolism. Recently, it has been shown that breakdown of storage triacylglycerol is an essential step for light induced stomatal opening, and the free fatty acids generated may be utilized for energy production to promote stomatal opening (McLachlan et al., 2016). In this study, an initial increases of free fatty acids and monoglycerol lipids were observed under low CO_2_ induced stomatal opening (Figures 3, 4), and two GDSL esterase/lipases increased concomitantly (Supplementary Table S3). These data suggest that the breakdown of storage lipids in response to low CO_2_ may function similarly as light induced stomatal opening. Additionally, membrane expansion is needed for the stomatal opening process, thus the generated free fatty acids may also be utilized for new membrane lipid synthesis. Furthermore, the increase of two Ras-related proteins (both 1.3-folds, with *p*-values of 0.04) at 5 min and 10 min of low CO_2_ treatment indicates involvement of membrane signaling. Roh family members of the Ras-related G proteins (ROPs) were found to be involved in endocytosis processes during ABA and high CO_2_ induced stomatal closure, and active ROP2 GTPase caused inhibition of ABA and elevated CO_2_ induced stomatal closure (Hwang et al., 2011). Thus, the increase of Ras-related proteins could promote stomatal opening, and this hypothesis needs to be further tested.

**Figure 7:**
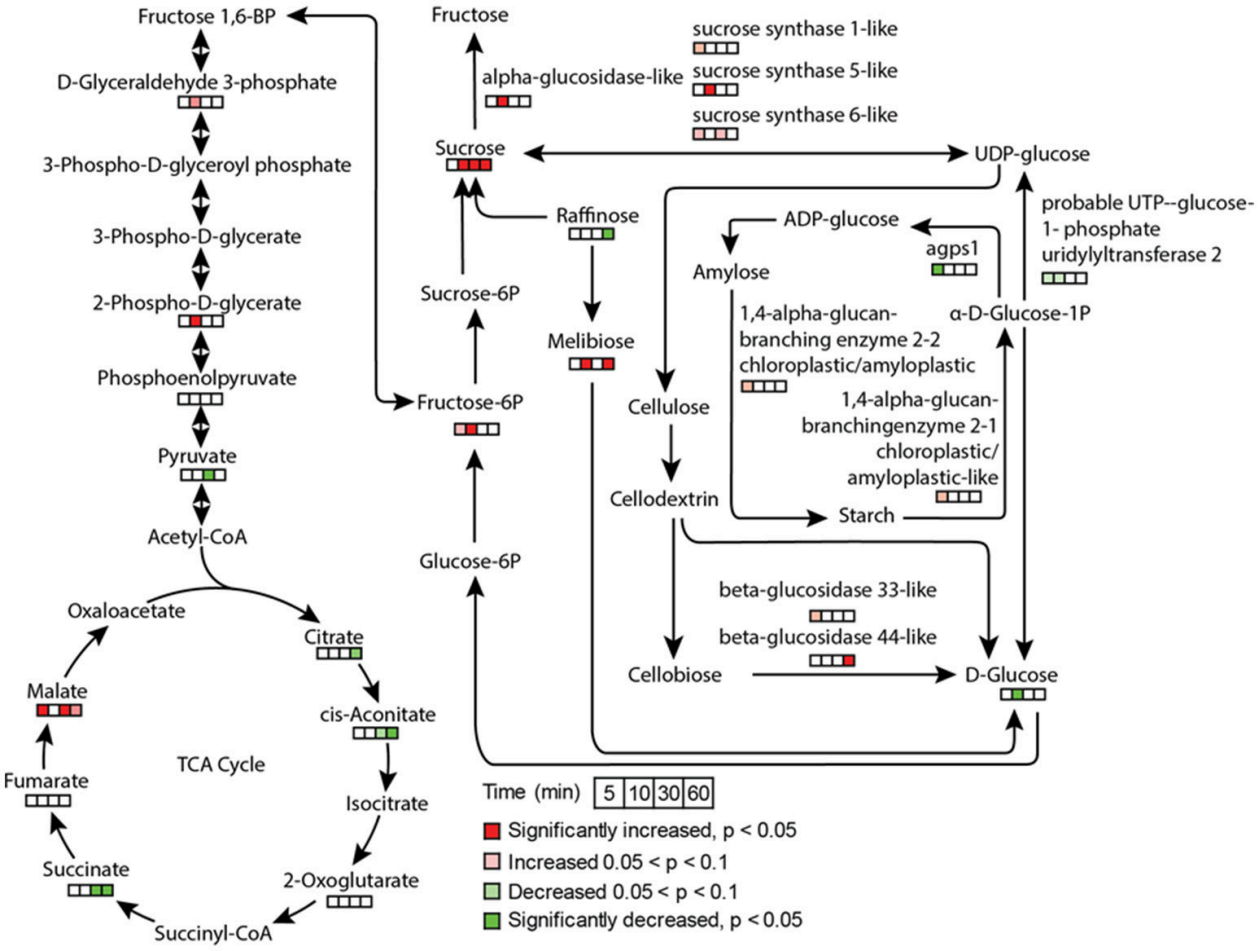
Metabolite and protein changes in response to low CO_2_ treatment in glycolysis, TCA cycle, and sucrose and starch metabolism. The pathways were drawn based on KEGG pathway database. The metabolites and proteins detected were indicated by time course square bars right below the molecules.

Stomatal opening requires energy, which can come from photosynthesis or respiration. Although the importance of guard cell photosynthesis has been under debate (Santelia and Lawson, 2016), our metabolomics data suggests significant contribution of glycolysis, starch and sucrose metabolism, and pentose phosphate pathway to low CO_2_ induced stomatal opening (Figures 3, 7). GO term enrichment of significantly changed proteins indicates significant changes in glycolysis pathway (Supplementary Figure S1). Proteins involved in glycolysis and TCA cycle, including glycerol-3-phosphate dehydrogenase, fructose-bisphosphate aldolase, enolase/phosphopyruvate hydratase, aconitate hydratase, and pyruvate dehydrogenase E1 component subunit alpha, all increased at both early and later time points (Supplementary Tables S2, S3). A recent study using chlorophyll-less guard cells showed that guard cell CO_2_ response does not depend on photosynthesis (Azoulay-Shemer et al., 2015), indicating respiration may be the main source of energy production during CO_2_ induced stomatal movement.

### Phytohormone crosstalk mediates low CO_2_ induced stomatal opening

Under low CO_2_, many phytohormones that induce stomatal opening may orchestrate to facilitate the opening process. Auxins were found to counteract ABA induced stomatal closure by preventing ROS and NO production (She and Song, 2006; Song et al., 2006). Cytokinins can scavenge the ROS and NO present in the cells, as well as prevent further ROS and NO production (She and Song, 2006; Song et al., 2006). Brassinosteroid (BR), on the other hand, can induce stomatal opening or closing depending on the concentration. A BR deficient mutant was shown to be hypersensitive to ABA induced stomatal closure (Ephritikhine et al., 1999), indicating BR also counteracts ABA. It has become clear that low concentrations of BR induce stomatal opening, while high concentrations can cause stomatal closure (Xu et al., 1994a,b; Haubrick et al., 2006). As expected, hormones known to induce stomatal opening, e.g., auxins, cytokinins, BR, gibberellins all showed increasing trends under low CO_2_. In addition, melatonin was also found to increase at 5 min and 10 min of low CO_2_ treatment (Figure 6).

Previously, we have found that JAs were involved in high CO_2_ induced stomatal closure (Geng et al., 2016). Under low CO_2_, we did not observe any significant changes of JAs (Figure 5). Instead, traumatic acid was found to be increased, implying its potential involvement in low CO_2_ induced stomatal opening. Work on drought stress in rice confirmed the competition of traumatic acid production with JA production (Liu et al., 2012). Similar to JAs, ABA levels did not show significant changes despite the increases of ABA catabolic products phaseic acid and dihydrophaseic acid under low CO_2_ treatment. These data suggest that low CO_2_ induced stomatal opening is a multihormone response. How the recently discovered metabolites (e.g., melatonin and traumatic acid) function in the guard cell low CO_2_ response and how they crosstalk with other hormones deserve further studies.

### Comparison between high CO_2_ and low CO_2_ responsive metabolites in guard cells

Here we compare the metabolite changes under low CO_2_ (this study) and high CO_2_ (Geng et al., 2016) to better understand CO_2_ regulation of guard cell metabolism and signaling. Interestingly, the changes in many different groups of metabolites, especially phytohormones, flavonoids, sugars and sugar alcohols showed opposite trends under the two CO_2_ treatment conditions (Supplementary Figure S2) (Geng et al., 2016). Phytohormones known to induce stomatal opening, including auxin, cytokinin and gibberellin all showed increases under low CO_2_ but not under elevated CO_2_. On the other hand, phytohormones that promote stomatal closure, such as ABA and JAs showed increases under elevated CO_2_ (Geng et al., 2016), but no significant changes under low CO_2_ (see previous section). In addition, we also found significant increases of melatonin at 5 min and 10 min of low CO_2_ treatment, and significant decreases under elevated CO_2_ (Figure 6, Supplementary Figure S2). Treatment with melatonin can inhibit plant ABA response by attenuating the ROS production, as well as repressing the expression of ABA biosynthesis genes and activating ABA catabolic genes (Li et al., 2015). How melatonin functions in guard cell CO_2_ responses is a very interesting question for future studies.

Two groups of flavonols, quercetins, and kaempferols were transiently increased at 5 min and 10 min under elevated CO_2_ (Geng et al., 2016). However, under low CO_2_, both metabolites showed decreases (Supplementary Table S1). Since flavonols have been indicated to be ROS scavengers, the decreases of antioxidants correlate well with the notion of minimal ROS production in guard cells under low CO_2_. Then how were ROS levels controlled under low CO_2_ conditions? At the proteome level, we observed that the significantly changed proteins were enriched in cell redox homeostasis processes (Supplementary Figure S1). These proteins include four peroxidases, a superoxide dismutase, a peroxisomal ascorbate peroxidase, two NADH-ubiquinone oxidoreductases, a sulfate reductase, a sulfate oxidase, and others involved in various cellular redox regulation processes. Most of the proteins such as peroxidases showed significant increases (Supplementary Table S3), suggesting their function of maintaining low levels of ROS to promote stomatal opening under low CO_2_. Consistent with this interpretation, metabolites related to ascorbate-glutathione cycle (e.g., dehydroascorbic acid and glutathione) also showed significant increases when compared to guard cells treated with elevated CO_2_ (Supplementary Figure S2).

As to sugars, sugar alcohols and organic acids, which are important for osmoregulation and stomatal opening, most displayed increases under low CO_2_, and no changes or decreases under elevated CO_2_ (Supplementary Figure S2). For example, sucrose and mannitol showed significant increases throughout the low CO_2_ treatment, and decreases under elevated CO_2_ (Figure 7). In pathway enrichment analysis, significantly changed metabolites were enriched in starch and sucrose metabolism and glycolysis/gluconeogenesis pathways (Figure 3). Together with the protein result that significantly changed proteins were overrepresented by the glycolysis pathway (Figure 7), we conclude the production of osmolytes to increase guard cell turgor pressure under low CO_2_ is mainly from starch breakdown and cellular respiration. Please note that not all the metabolites showed opposite patterns of changes under low CO_2_ and elevated CO_2_ conditions. For example, malate levels increased under both elevated CO_2_ and low CO_2_. The sources of malate may be different under these two conditions. Since guard cells have a similar pathway of incorporating CO_2_ as C4 photosynthesis (Willmer et al., 1973), it is highly possible that a temporal elevation of CO_2_ increases malate through the activities of phosphoenolpyruvate carboxylase and NADP-malate dehydrogenase (Willmer et al., 1973; Asai et al., 2000). The result of malate increase contradicts with previous findings on stomatal closure induced by other stimuli such as darkness (Penfield et al., 2012), elevated CO_2_ (Lee et al., 2008) and ABA (Asai et al., 2000). It is possible that malate may not function as an intracellular osmolyte under elevated CO_2_ (Geng et al., 2016). While under low CO_2_, malate may be imported or produced through starch degradation to function as a major osmolyte and counter ion for K^+^ (Santelia and Lawson, 2016).

## Conclusions

Using hyphenated metabolomics and 10-plex TMT based proteomics technologies, we have investigated the metabolomic and proteomic responses of *B. napus* guard cells to low CO_2_. A total of 411 metabolites and 1,397 proteins were quantified in this time-course study. Metabolites and proteins that exhibited significant changes are overrepresented in fatty acid metabolism, hormone metabolism, starch and sucrose metabolism, glycolysis and redox regulation. Compared to the previous elevated CO_2_ study (Geng et al., 2016), many interesting changes were noted in guard cells under low CO_2_. For example, diversion of JA biosynthesis to traumatic acid biosynthesis, the role of melatonin and phytohormone crosstalk, redox regulation and the functions of fatty acid metabolism and Ras-related protein. Future studies focusing on testing the hypotheses generated from this work will greatly enhance our understanding of the molecular mechanisms underlying low CO_2_ induced stomatal opening. In addition, as the MS imaging technique moves toward high resolution, *in situ* guard cell metabolomics will be an exciting direction.

## Author contributions

SG performed the metabolomics and proteomics experiments, data analysis and paper drafting; BY collected experimental materials and performed proteomics experiments; NZ participated in peptide labeling and data analysis; CD contributed in LC-MS optimization and TMT peptide analysis; and SC designed the experiments, supervised the work and finalized the manuscript. All the authors have read the manuscript and provided comments.

### Conflict of interest statement

CD is associated with Thermo Scientific Inc., and there is no conflict of interest in this basic research. CD contributed in LC-MS optimization for achieving optimal resolving power of the 10-plex TMT tagged peptides and acquired the LC-MS data of the TMT peptides that used to identify the low CO_2_ responsive proteins in this study. The other authors declare that the research was conducted in the absence of any commercial or financial relationships that could be construed as a potential conflict of interest.

## Supplementary material

Supplementary Figure S1 Protein single enrichment analysis of proteins changed in guard cells. **(A)** Biological process; **(B)** Molecular function. The GO terms in the boxes are labeled by GO ID, term definition and statistical information. The significant terms (*p* < 0.05) are marked with color, while non-significant terms are shown as white boxes. Solid, dashed, and dotted lines represent two, one and zero enriched terms at both ends, respectively. The enrichment level is related to the term *p*-values, i.e., the smaller of the term's *p*-value, the more significant statistically, and the node's color is darker and redder. Inside the box of the significant terms, the information includes: GO term, *p*-value, GO description, item number mapping the GO in the query list and background, and total number of query list and background. For those terms with *p*-values > 0.05, only GO information is shown.

Supplementary Figure S2 Changes of osmoregulators, ROS scavengers, phytohormones and melatonin in guard cells under elevated CO_2_ (red) and low CO_2_(cyanine blue) conditions. Significant metabolite changes between elevated and low CO_2_ treatments (*p* < 0.05) are marked with stars (∗).

Supplementary Table S1 Metabolite information, quantification, mean, fold change, and *p*-value. Identification levels include: (1) Identified compounds; (2) Putatively annotated compounds; (3) Putatively characterized compound classes; (4) Unknown compounds for compound identification levels as recommended by Metabolomics Society's metabolomics standards initiative (MSI).

Supplementary Table S2 List of all the identified peptides and proteins in the two independent experiments. Highlighted cells indicate proteins with a significance level of *p* < 0.05.

Supplementary Table S3 List of all the significantly changed proteins (fold change < 0.8 or > 1.2, *p*-value < 0.05) at different time points, with *p*-value and fold change at their corresponding time points.
