# Supplementary material for: Metabolomics and Proteomics of Brassica napus Guard Cells in Response to Low CO2
Source: Front Mol Biosci. 2017 Jul 25;4:51. doi: 10.3389/fmolb.2017.00051 (PMC5525006; doi:10.3389/fmolb.2017.00051)
Supplement: Supplementary Figure S1 — Protein single enrichment analysis of proteins changed in guard cells. (A) Biological process; (B) Molecular function. The GO terms in the boxes are labeled by GO ID, term definition and statistical information. The significant terms (p < 0.05) are marked with color, while non-significant terms are shown as white boxes. Solid, dashed, and dotted lines represent two, one and zero enriched terms at both ends, respectively. The enrichment level is related to the term p-values, i.e., the smaller of the term's p-value, the more significant statistically, and the node's color is darker and redder. Inside the box of the significant terms, the information includes: GO term, p-value, GO description, item number mapping the GO in the query list and background, and total number of query list and background. For those terms with p-values > 0.05, only GO information is shown. [file Image1.PDF]

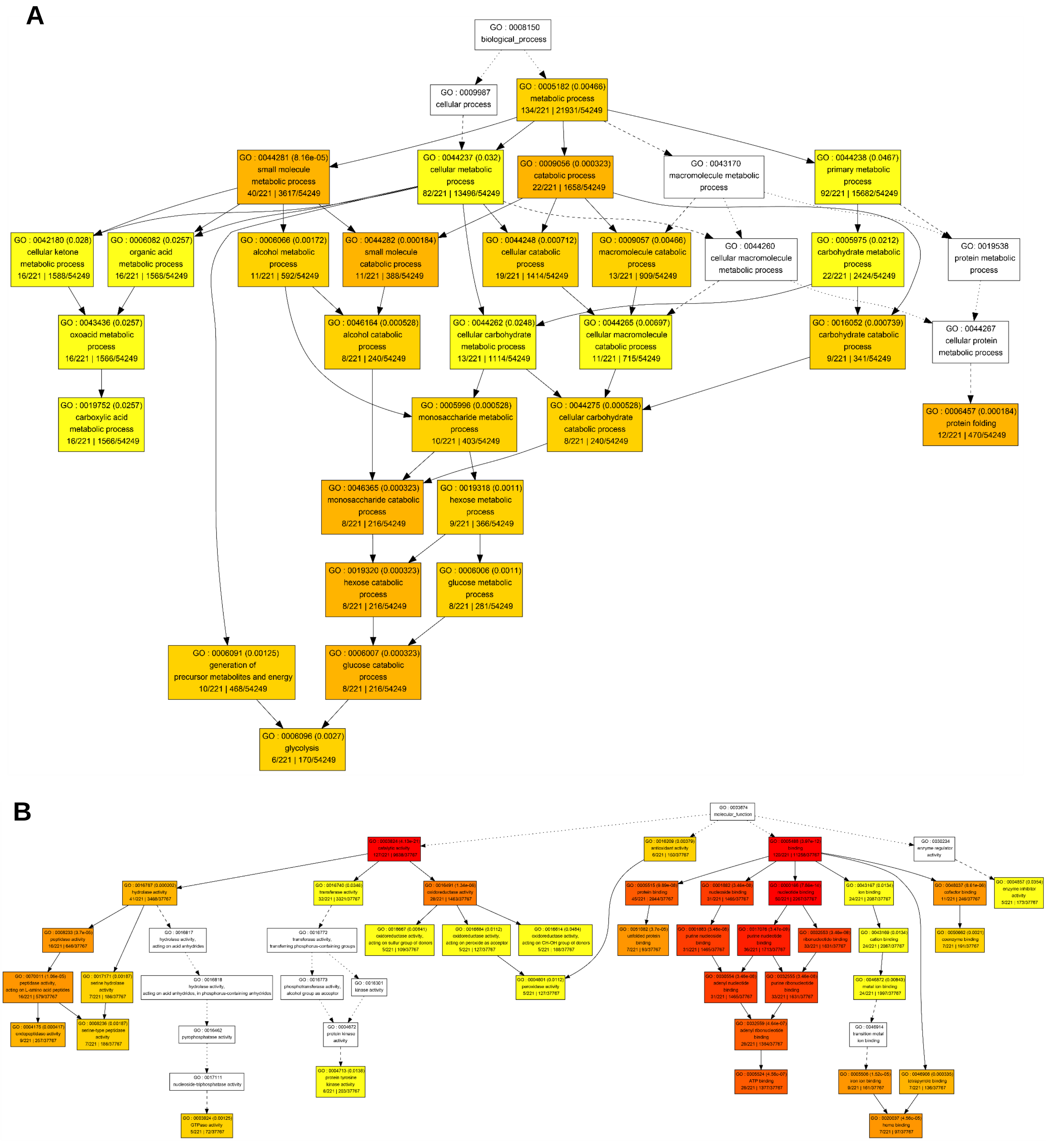

Supplemental Figure 1
