# Supplementary figures and images for: Metabolomics and Proteomics of Brassica napus Guard Cells in Response to Low CO2
Source: Front Mol Biosci. 2017 Jul 25;4:51. doi: 10.3389/fmolb.2017.00051 (PMC5525006; doi:10.3389/fmolb.2017.00051)

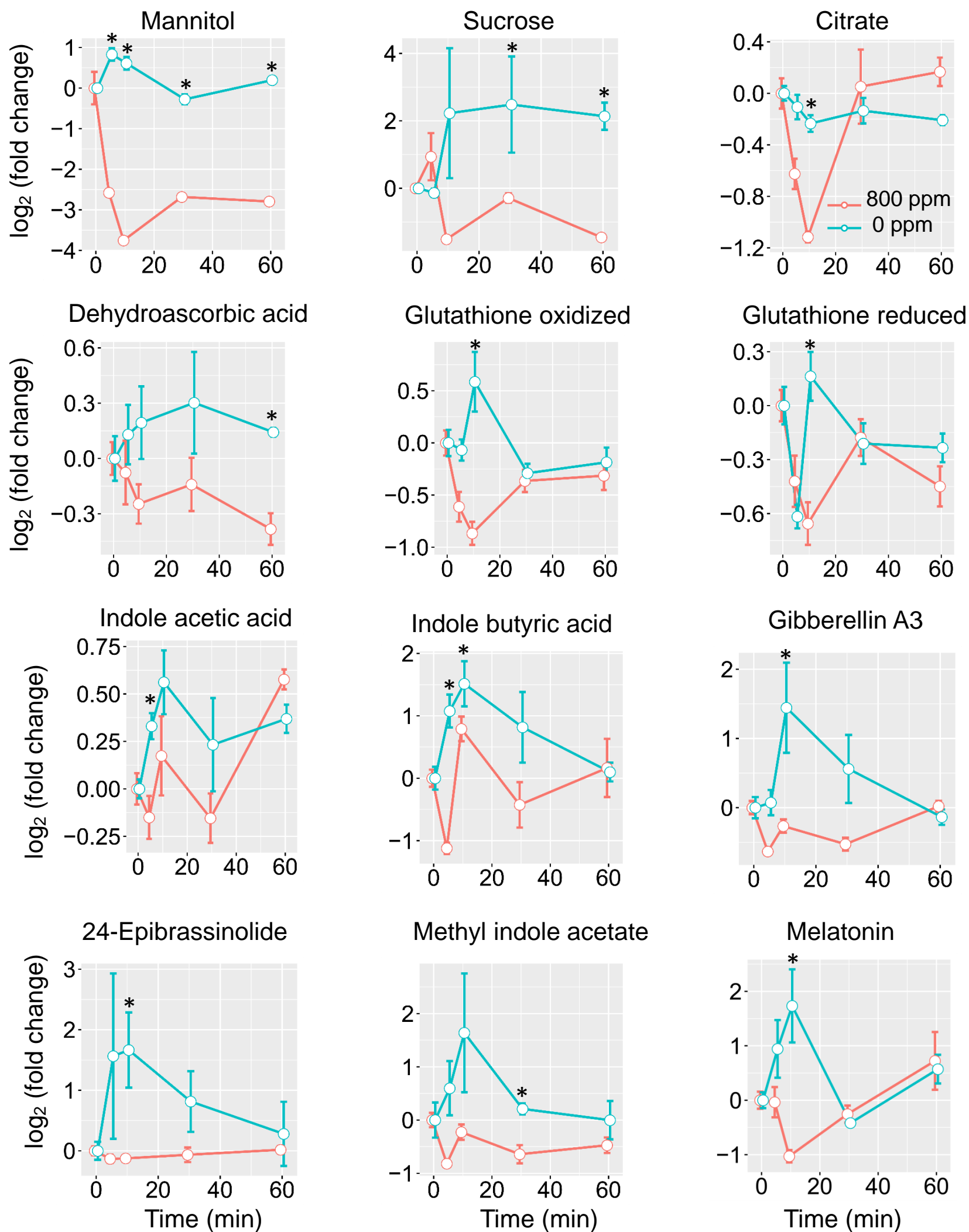

Supplemental Figure 2

Supplement: Supplementary Figure S2 — Changes of osmoregulators, ROS scavengers, phytohormones and melatonin in guard cells under elevated CO2 (red) and low CO2(cyanine blue) conditions. Significant metabolite changes between elevated and low CO2 treatments (p < 0.05) are marked with stars (∗). [file Image2.PDF]
